# Imaging beyond ultrasonically-impenetrable objects

**DOI:** 10.1038/s41598-018-23776-7

**Published:** 2018-04-10

**Authors:** Tali Ilovitsh, Asaf Ilovitsh, Josquin Foiret, Katherine W. Ferrara

**Affiliations:** Department of Biomedical Engineering, University of California, Davis, California USA

## Abstract

Ultrasound images are severely degraded by the presence of obstacles such as bones and air gaps along the beam path. This paper describes a method for imaging structures that are distal to obstacles that are otherwise impenetrable to ultrasound. The method uses an optically-inspired holographic algorithm to beam-shape the emitted ultrasound field in order to bypass the obstacle and place the beam focus beyond the obstruction. The resulting performance depends on the transducer aperture, the size and position of the obstacle, and the position of the target. Improvement compared to standard ultrasound imaging is significant for obstacles for which the width is larger than one fourth of the transducer aperture and the depth is within a few centimeters of the transducer. For such cases, the improvement in focal intensity at the location of the target reaches 30-fold, and the improvement in peak-to-side-lobe ratio reaches 3-fold. The method can be implemented in conventional ultrasound systems, and the entire process can be performed in real time. This method has applications in the fields of cancer detection, abdominal imaging, imaging of vertebral structure and ultrasound tomography. Here, its effectiveness is demonstrated using wire targets, tissue mimicking phantoms and an *ex vivo* biological sample.

## Introduction

Non-invasive, real-time ultrasound imaging is a powerful diagnostic tool with advantages including cost effectiveness, deep penetration, widespread availability and safety. In particular, ultrasound has been used as a screening tool for breast cancer due to its ability to differentiate between benign cysts and malignant tissue. Such differential diagnosis requires a thorough analysis of the margin of the lesion and its shape and echo properties^1–3^. To reduce false detection rates, ultrasound imaging is frequently used as a follow-up technique to mammography. Sonographic evaluation of lesions elsewhere in the body is, however, often restricted by the presence of objects with a significant impedance difference along the ultrasound path, hampering the access to deeper structures. Bones, calcifications, and trapped air bubbles within the abdomen and the lungs, for example, are obstacles to ultrasound waves. Interrogation of targets that lay behind these impermeable obstacles is problematic. A similar challenge exists in high intensity focused ultrasound therapy^4^, where heat generated by ultrasound propagation is used to ablate a small volume of tissue in order to treat diseases, including cancer and neurological disease, in a minimally-invasive manner^5^. In abdominal applications, achieving a specified therapeutic dose at the focus while minimizing undesired heating of structures in the ultrasound path (e.g. the rib cage) can be difficult.

Various techniques have been used to overcome the difficulties associated with ultrasound imaging and therapy in the presence of obstacles. One solution is to use a geometrical approach to turn off transducer elements for which energy will primarily be absorbed by the obstacle^6^. Other methods combine the geometrical approach with improved focusing^7,8^ and beam shaping^9^. Because these techniques have been applied for therapeutic purposes, they have relied on continuous wave insonation, rather than on single-cycle transmission (as is required for imaging purposes). In the context of bypassing obstacles for imaging applications, when the obstacle is positioned in close proximity to the array such that elements are blocked or are non-functioning, the redundancy in the round-trip ultrasound signal can be used in post processing to compensate for the missing signals and recover full resolution^10^. This redundancy method is geared toward solving problems resulting from thin obstacles on or near the transducer surface itself (such as malfunctioning elements). If the problem involves a thick obstacle farther from the transducer, the redundancy method cannot be used. Another method uses the geometrical approach combined with optimization based on the pseudo-inverse (PI) method^11^ to adapt focusing in imaging applications^12^. The PI method, however, requires the computation of an analytical solution to the inverse problem, hence its implementation is not simple. The performance of the PI method is compared to our proposed method in the simulation and experimental results section below.

**Figure 1:**
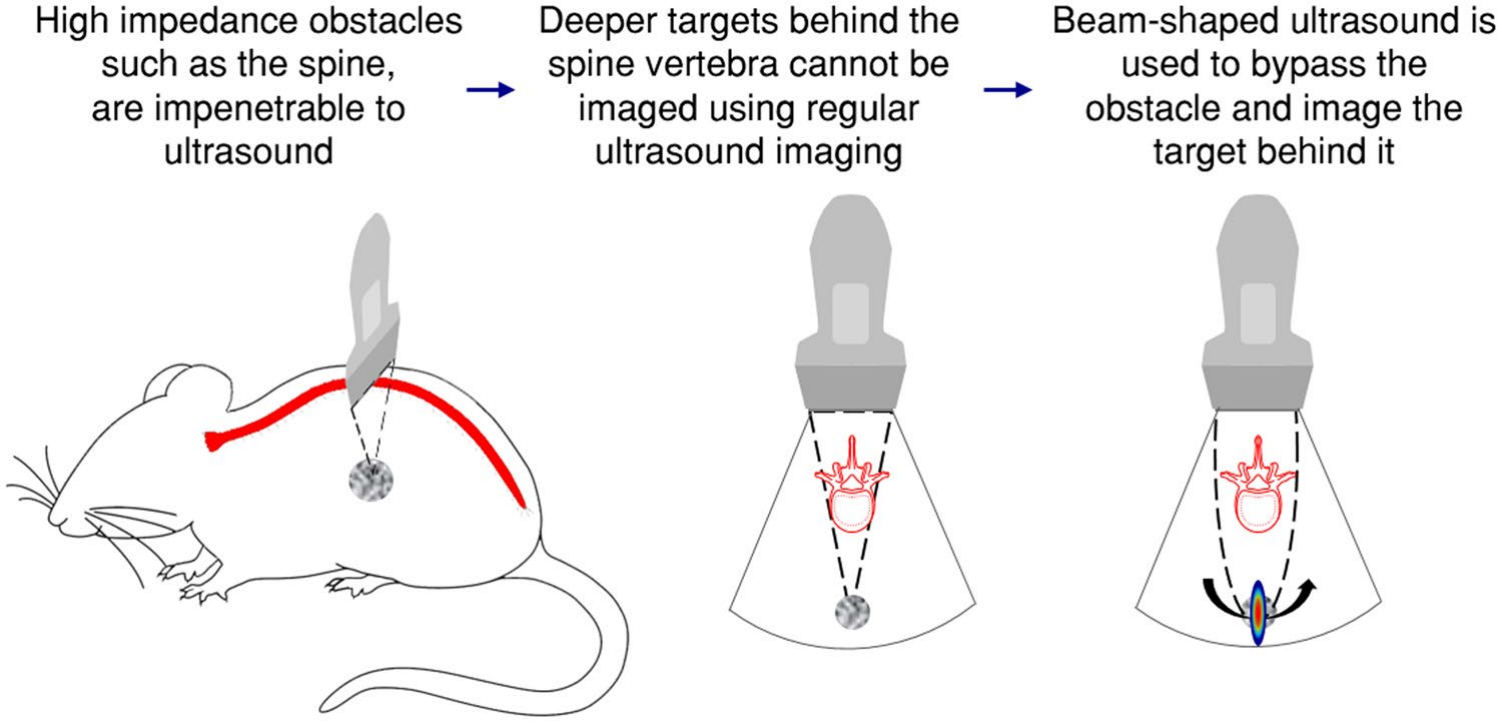
Schematic illustration of the BUS method. When a bone obstacle such as the spine is located in front of an object, the emitted field cannot penetrate the obstacle and image the deeper targets. Ultrasound beam shaping technology is used to design a field pattern that bypasses the obstacle and focuses at the object behind it. The imaging process is similar to two-way focusing in that the focal point is steered across the target. The set of captured images is post processed to reconstruct an image in which the object is visible.

Our goal is to develop and implement a method to improve imaging beyond impenetrable obstacles in pulse-echo acquisition. Here, we propose applying beam-shaping technology to manipulate the acoustic wavefront in order to look behind solid objects that would otherwise be impenetrable to ultrasound. We call this method “bypassing ultrasound” (BUS), and the BUS method is illustrated in Fig. 1. Beam shaping is achieved by controlling the phase and apodization of each individual transducer element, providing the method with the distinct advantage of being dynamic and reconfigurable in real-time. Ultrasound beam shaping is often used in hyperthermia treatments^13,14^, ultrasonic neuro-modulation^15^, and in the generation of acoustic holograms^16^. Recently, we applied beam shaping to super-resolution ultrasound imaging^17^. Other beam shaping algorithms include the PI method^11^ mentioned above, the conjugate field method^13^ and the Gerchberg–Saxton phase retrieval algorithm^18^. Among these methods, the Gerchberg–Saxton algorithm yields the best results in terms of efficiency and focal spot uniformity^15^.

The transmitted beam patterns applied in our imaging studies include a gap at the obstacle position (hence the field bypasses the obstacle) and a focal point at the target position. A B-mode image of the sample provides feedback from which the obstacle width and position and the focal position can be estimated by the user. Image acquisition is then similar to standard two-way focusing^19^. On transmission, each element is excited with a single cycle pulse. The Gerchberg-Saxton algorithm determines the time delays for each of the elements, and focuses the transmitted ultrasound beam to a specific point. Formation of a complete ultrasound image requires the steering of this focal point across the target using multiple transmit-receive events, typically 50 events will suffice. The returning echoes from these multiple transmissions are combined to reconstruct a complete ultrasound image.

## Theoretical Background

### Design and validation of beamforming technique

**Figure 2:**
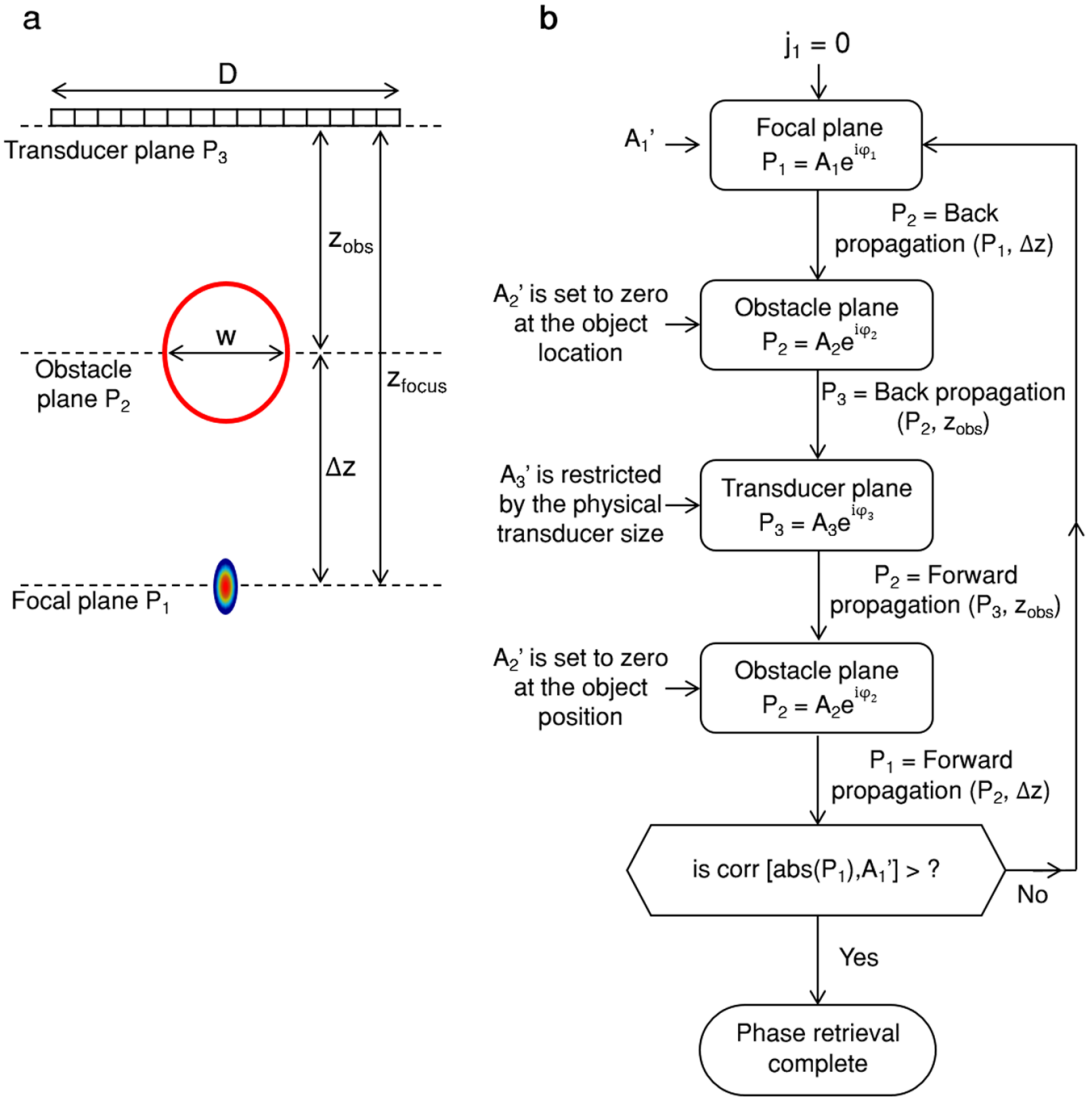
Pattern design illustration. (**a**) Schematic representing ultrasound imaging of an object behind an impenetrable obstacle. There are three planes of interest: the transducer plane, the obstacle plane, and the focal plane, which have acoustic fields of P_1_, P_2_ and P_3_, respectively. An obstacle with a width w is located at a distance of z_obs_ from the transducer. The goal is to generate a focal spot at the target location, which is positioned at a distance Δz from the obstacle. (**b**) Flowchart of the modified Gerchberg–Saxton process designed to create a focal point behind an obstacle.

The iterative Gerchberg–Saxton phase retrieval algorithm is designed to retrieve the phase of a propagating field from a set of imaging planes related via a propagation function. Ultrasound requires near-field propagation, which can be computed with low computational cost using the angular spectrum method^20^. The algorithm iteratively propagates the acoustic wave backward and forward between three planes; the focal plane, the obstacle plane and the transducer plane (as schematically illustrated in Fig. 2a). The algorithm iterates until the correlation between the computed amplitude and the desired amplitude reaches a predetermined threshold, yielding the phase and apodization distributions of the transducer that will generate the designed emitted field. Expressed mathematically, if P(x, z) denote the complex harmonic pressure at a single frequency in a uniform medium, the acoustic field is expressed as:

1 $$P(x,\,z)=A(x,z){e}^{j{\rm{\phi }}(x,z)}$$

where A and φ are the amplitude and phase terms, respectively. In Fig. 2a, P_1_ is the pressure at the focal plane, P_2_ is the pressure at the obstacle plane and P_3_ is the pressure at the transducer plane. The aim of the algorithm is to determine the field at the transducer plane that will maximize the intensity at the focal plane. To do this, the geometrical parameters of the distance between the obstacle and transducer (z_obs_), the distance between focal spot (target location) and the transducer (z_focus_), the distance between the target and the obstacle (Δz), the width of the obstacle (w), and the transducer aperture width (D), must be defined. These parameters are estimated from a standard B-mode image of the sample.

A_1_, A_2_ and A_3_ are binary amplitude vectors, where each point in a vector corresponds to a spatial location along the beam path. A_1_ is the amplitude at the focal plane, where a single focal point is desired. A_1_’ is the ideal amplitude at the focal plane, with elements equal to zero except for a single position at the focal spot equal to one. A_2_ is the amplitude at the obstacle plane. A_2_’ is the ideal amplitude at the obstacle plane, where the vector elements that correspond to the location of the obstacle with a width w are set to zero. A_3_ is the amplitude at the transducer plane. A_3_’ is the ideal amplitude at the transducer plane, and is restricted by the transducer’s physical dimensions. The vector elements that correspond to spatial locations outside of the transducer aperture are set to zero. Additionally, in order to maximize the transmitted intensity, the vector elements in A_3_’ that correspond to the aperture spatial location are set to one.

A flowchart of the method is presented in Fig. 2b. In the first iteration, P_1_ is composed of the ideal amplitude A_1_’ and a zero phase φ_1_. P_1_ is then back-propagated from the focal plane to the obstacle plane using the angular spectrum method. The result is the amplitude and phase that define P_2_. The elements in A_2_ that correspond to the spatial location of the obstacle are set to zero, and the calculated phase φ_2_ is maintained. P_2_ is back propagated to the transducer plane. In P_3_, the ideal amplitude A_3_’ is imposed, and the calculated phase φ_3_ is maintained. P_3_ is forward propagated to P_2_. Again, the ideal amplitude of A_2_’ is imposed, and the calculated phase φ_2_ is retained. Next, P_2_ is forward propagated to P_1_. The algorithm calculates the correlation between the obtained amplitude of P_1_, and the ideal amplitude A_1_’. If the correlation is higher than a predetermined threshold, the process is finished. If the correlation is below the threshold, the ideal amplitude of A_1_’ is imposed, the calculated phase φ_1_ is retained, and this process continues to iterate until convergence. Typically, this process requires a few tens of iteration cycles for the correlation to reach a threshold of 0.95. Once the process is completed, the phase of P_3_ (φ_3_) is used to determine the delay for each of the transducer elements that maximizes the focal intensity.

**Figure 3:**
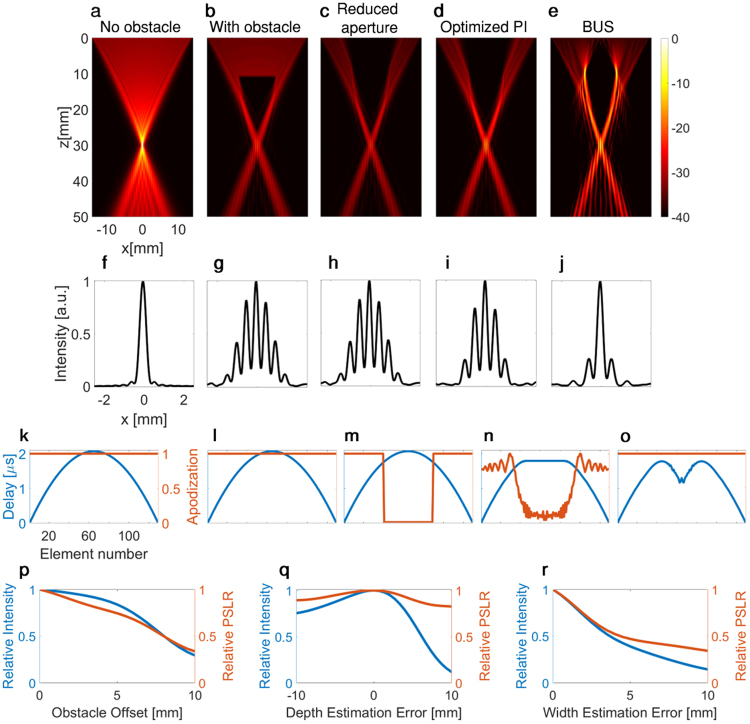
Comparison of the emitted acoustic intensity fields for the various imaging methods for a transducer aperture width (D) of 28.2 mm. Axes are common to (**a**–**e**),(f–j),(k–o). (**a**–**e**) are presented with a 40-dB dynamic range and a common colorbar (**a**) Emitted field for two-way focusing with focus at a depth of z = 30 mm (without the presence of an obstacle). (**b**–**e**) intensities are normalized with respect to (**a**). (**b**) Emitted field for two-way focusing focused at a depth of z = 30 mm, in the presence of a 10-mm diameter obstacle located at z = 10 mm. (**c**) Emitted field for two-way focusing where elements are turned off when the obstacle is in the direct path of the ray’s elements. (**d**) Optimized PI method. (**e**) BUS method designed to bypass the obstacle. (**f**–**j**) Lateral intensity profiles at the focus (z = 30 mm) for (**a**–**e**), respectively. (**k–o**) Delay (blue) and apodization (red) maps used for (**a–e**), respectively. (**p**) Effect of obstacle offset from the transducer center line on the focal intensity and peak-to-side-lobe ratio (PSLR). (**q**) Effect of depth estimation error on the focal intensity and PSLR. (**r**) Effect of width estimation error on the focal intensity and PSLR.

While the width and depth of the obstacle are pre-determined for a specific application, there are tradeoffs in the selection of the transducer aperture width and the focal location (Δz from the obstacle). A dead zone exists beyond each obstacle, in which a focus cannot be created, and its size determines the minimum distance between the obstacle and the focus (Δz_min_). In typical two-way focusing protocols in the absence of the obstacle, when the entire aperture is transmitting, the ray-trace cone is focused to a point in the focal plane. Figure 3a is a simulation that shows the emitted field propagation focused at a depth of z = 30 mm. The transducer parameters match the phased array sector transducer P6-3 (ATL Ultrasound Inc., Bothell, WA, USA) used in most of our experiments, which has D = 28.2 mm. This result is used as the reference for all other results, and the intensities are normalized accordingly. When an obstacle is present along the ultrasound propagation path, a portion of the beam is blocked such that the generated focal spot is substantially reduced in intensity and distorted due to higher sidelobes. We focus on the case where the obstacle blocks roughly one third of the transducer (w/D = 1/3).

We compared the focal spot generated behind a 10-mm obstacle located at a depth of 10 mm (z_obs_ = 10 mm) using various beamforming methods. When standard two-way focusing is applied, the intensity at the focal point is reduced by a factor of 10 (Fig. 3b) compared to the ideal case where no obstacle is present. To reduce undesired reflections from the obstacle, it is possible to turn off the elements in front of the obstacle (Fig. 3c). However, the focal point intensity and profile remain similar to those resulting from standard two-way focusing. The optimized PI method improves the focal intensity by a factor of 2 compared to standard two-way focusing (Fig. 3d), however, the intensity is reduced by a factor of 5 compared to the case where no obstacle is present. Using the BUS method (Fig. 3e), the intensity at the focal point is one-half of the reference case, an improvement of a factor of 5 compared to both two-way focusing methods, and an improvement by a factor of 2.5 compared to the optimized PI method.

For each of the five methods introduced in Fig. 3a–e, the point spread function is summarized in Fig. 3f–j, respectively. The width of the main lobe is similar for each case. The lateral resolution for each of the discussed methods remains similar to that achieved without the obstacle, since the width of the transmit aperture is unchanged^10^. Elevated sidelobes (with a maximal intensity of 0.8) are apparent in the presence of the obstacle in Fig. 3g–i. The sidelobes are reduced to a maximal value of 0.25 with the BUS method (Fig. 3j), therefore the peak-to-side-lobe ratio (PSLR) obtained with the BUS method is higher compared to the other methods. Further, Fig. 3k–o present the delay and apodization maps that were applied to generate the emitted fields in Fig. 3a–e, respectively.

The BUS method is designed to image targets distal to an obstacle. A focal point is also generated when the obstacle is offset from the transducer center line; however, its maximal intensity and PSLR are reduced compared to the no offset case (Fig. 3p). At an offset of 4 mm, the relative maximal intensity is reduced by 10% and the relative PSLR by 20%. This decrease of 10% in the intensity was chosen here as a tolerated error that yields a useful beam.

Since the method requires user feedback to determine the obstacle’s width and depth, the effect of an uncertainty in these parameters on the focal intensity and PSLR was investigated for the same geometry discussed in Fig. 3. In each simulation, an error in the obstacle width or depth was introduced and the maximal intensity and PSLR were evaluated compared to the ideal case without estimation error. An error in the depth is more significant when the obstacle depth is underestimated (i.e. the obstacle is deeper than the estimation). An underestimation of 3 mm reduces the relative maximal intensity by 10% and the relative PSLR by 5% (Fig. 3q). An underestimation of the obstacle is more significant than overestimation of the obstacle depth (i.e. the obstacle is closer to the transducer than the estimation), as can be seen from the asymmetrical behavior of the graph. The most significant error results from underestimation of the obstacle width. For this case, an underestimation of 1 mm reduces the relative maximal intensity by 10% and the relative PSLR by 5% (Fig. 3r).

**Figure 4:**
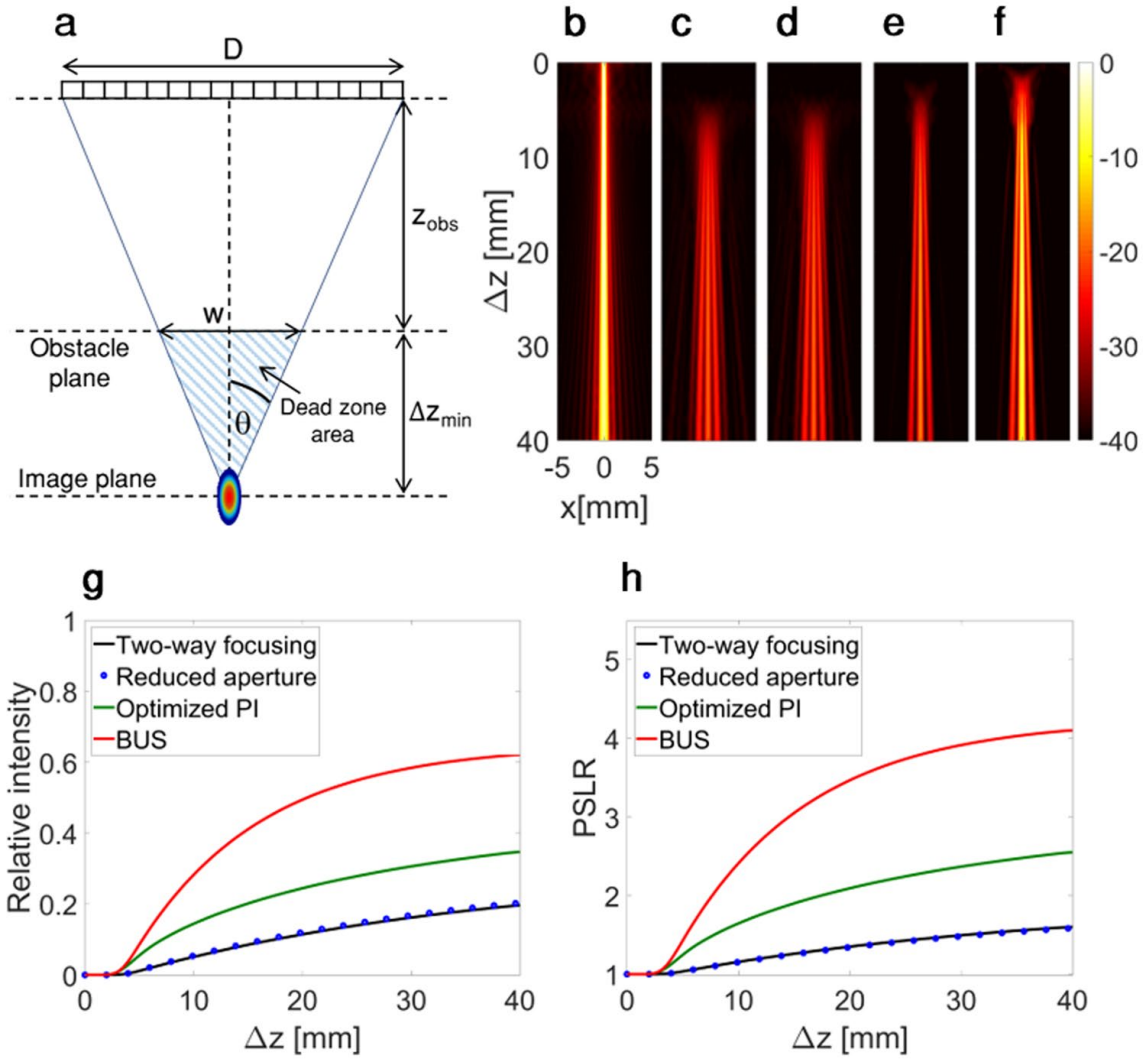
Spatial variation in ultrasound as a function of the axial separation of the obstacle and target (Δz), for transducer aperture width (D) of 28.2 mm. Axes are common to (**b–e**) and are presented with a 40 dB dynamic range with a common colorbar. The intensity is normalized by that obtained with two-way focusing in the absence of the obstacle. (**a**) Geometry of the depth of the obstacle (z_obs_), aperture (D), width of the obstacle (w), and minimum axial separation of the target and obstacle (Δz_min_). (**b**–**e**) The field intensity as a function of depth and axial distance for: (**b**) Reference two-way focusing without an obstacle. (**c**) Two-way focusing in the presence of the obstacle. (**d**) Two-way focusing with reduced aperture. (**e**) Optimized PI method. (**f**) BUS method. (**g**) Center axial line intensity (x = 0) for (**b**–**f**). (**h**) Center axial line (x = 0) peak to sidelobe ratio (PSLR) for (**b**–**f**).

To quantify the minimal distance behind an obstacle at which a focus can be generated, a geometrical model is considered, as summarized in Fig. 4a. The limiting case is that for which the obstacle blocks the entire emitted field, and only the beam-shaped field that bypasses the obstacle arrives at the target. The dead zone size, where a target cannot be detected, corresponds to the striped triangular area and can be estimated based on triangle similarity:

2 $$\Delta {z}_{min}=\frac{w}{D}\times {z}_{obs}$$

The dead zone area (S) is described by:

3 $$S=w\times \Delta {z}_{min}=\frac{({w}^{2}{z}_{obs})}{D}$$

The dead zone size is therefore dependent on the width and depth of the obstacle (Eq. 2) and can be reduced by decreasing the obstacle’s width or depth or by increasing the size of the aperture. The minimum distance between the obstacle and a focal point (Δz_min_) is dependent on the aperture size and the width and depth of the obstacle.

In comparison to the other methods, the BUS method results in the generation of a higher-intensity focus closer to the obstacle. To evaluate the improvement, the intensity of the lateral point spread function as a function of the axial distance between the obstacle and the focal point (Δz), was simulated for each of the strategies presented in Fig. 3a–e. For each iteration, Δz was varied and the maximal line profile intensity at the focal spot was captured and combined into a 2D image. Figure 4b provides the beam intensity for two-way focusing in the absence of the obstacle and the resulting image intensities were normalized with respect to this image. Due to diffraction, the beam width broadens as a function of depth, hence the maximum intensity decreases with distance. For two-way focusing in the presence of the obstacle, when transmitting with all elements (Fig. 4c) or with a reduced aperture (Fig. 4d), the main lobe intensity is reduced and sidelobes are apparent. The optimized PI method enhances the main lobe intensity and reduces the sidelobes (Fig. 4e), however performance is most improved with the BUS method (Fig. 4f). To compare the peak intensity distal to the obstacle as a function of distance for the various methods, line profiles along the centerline (x = 0) for Fig. 4b–f are presented in Fig. 4g.

The focal intensity increases at a smaller axial separation distance between the obstacle and target using the BUS method, as compared with conventional imaging methods. For BUS, the minimum separation distance between the obstruction and the target (Δz_min_) is 3.7 mm; close to the theoretical value of dead zone size based on Eq. 2, which is 3.5 mm. For the standard two-way focusing methods when using either the entire aperture or the reduced aperture, this separation distance reaches 5.5 mm, a value that is 1.5-fold larger than with the BUS method. For the optimized PI method, this distance is 4.4 mm, which is 1.3-fold larger than the BUS method. The field intensity distal to the obstacle is also higher with the BUS method for all separation distances, with the improvement peaking at a factor of 5.

The PSLR as a function of the distance between the focus and the obstacle, is presented in Fig. 4h, reaching 4.1, 2.2, 1.6 for the BUS, PI and standard two-way focusing methods, respectively. This suggests that the BUS method is expected to have the highest contrast of the three methods.

In the presence of the obstacle, the standard two-way focusing method using all of the elements yields very similar results to the method where the elements in front of the obstacle are not utilized, hence in the following examples the comparison was performed only with two-way focusing using all elements.

### Effect of the width of the obstacle

**Figure 5:**
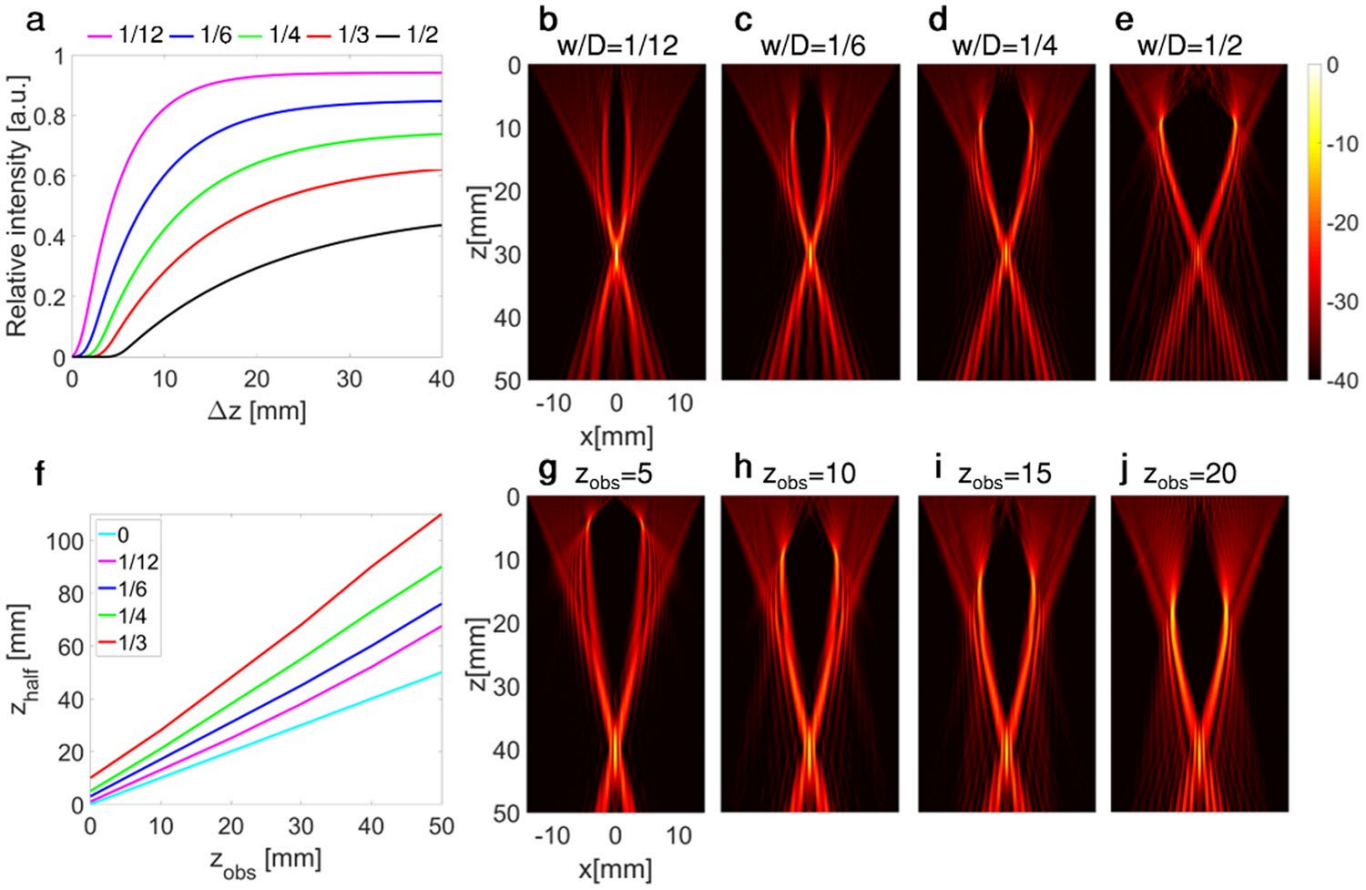
Simulated effect of the obstacle width and depth for D = 28.2 mm. (**a**) The focal point intensity as a function of the distance of the target from the obstacle for varied w/D, normalized by the intensity obtained with two-way focusing without the obstacle. (**b**–**e**) Emitted field for z_obs_ = 10 mm, z_target_ = 30 mm, and w/D = 1/12, 1/6, 1/4, 1/2 in b-e, respectively. (**f**) Depth at which the axial center line intensity reaches 1/2 of its maximal value (z_half_) as a function of the obstacle depth, for varied w/D. (**g**–**j**) Emitted field for z_obs_ of 5, 10, 15, 20 mm for (**g**–**j)**, respectively. Axes and 40 dB dynamic range colorbar are common to (**b**–**e**), and (**g**–**j**). For figures (**g**–**j**), w/D = 1/3 and z_target_ = 40 mm.

The intensity and spatial distribution of the ultrasound field behind the obstacle depend on the ratio of w/D and the location of the obstacle. In order to characterize the influence of the obstacle width on the focal intensity, the depth of the obstacle was fixed at 10 mm, and the transducer aperture at 28.2 mm. For each value of relative obstacle width, expressed as a fraction of transducer aperture (1/12, 1/6, 1/4, 1/3, 1/2), the focal peak intensity as a function of the depth of the target distal to the obstacle (Δz), as defined in Fig. 4a, was simulated using the BUS method. The results were normalized with respect to the reference case of  two-way focusing without an obstacle (Fig. 5a). These results confirm that increasing obstacle width decreases the maximum focal intensity. Furthermore, the slope of the graphs in Fig. 5a is steeper for a smaller w/D, i.e. a higher intensity focal point can be generated at closer distances. Figure 5b–e present the emitted field for fixed z_obs_ = 10 mm and z_target_ = 30 mm (i.e. Δz = 20 mm) for a relative width w/D of 1/12, 1/6, 1/4 and 1/2, respectively. It is important to note that while the intensity is decreased relative to the no-obstacle case, the BUS method performs better in the presence of an obstacle than other beamforming methods, achieving, for example, a 30-fold difference in the field intensity at the target site over two-way focusing for w/D = 1/2.

### Effect of the obstacle depth

Since the emitted field is focused to a single point, the ray trace cone is narrower near the focus. Therefore, for a deeper obstacle, a greater fraction of the emitted field is blocked. This suggests that the focal intensity depends both on the ratio z_obs_/z_focal_, and most significantly on the absolute position of the obstacle (z_obs_). To evaluate the effect of obstacle depth (z_obs_), simulations were conducted where the focal distance was plotted as a function of z_obs_ for each value of w/D (Fig. 5f). The focal distance was chosen as the depth where the focal region reaches half of its maximal intensity (z_half_). The results indicate that for a given ratio of z_obs_/z_focal_, a deeper obstacle will require a higher separation distance between the obstacle and the focus. Also, for a fixed focal distance, the focal intensity decreases when the obstacle approaches the focal location. This is visualized in Fig. 5g–j, where fixed parameters of w/D = 1/3 and z_target_ = 40 mm were chosen, and z_obs_ was varied between 5, 10, 15, and 20 mm in Fig. 5g–j, respectively. While the focal distance remained the same, the relative intensities were reduced as a function of obstacle depth and reached 71%, 60%, 50%, 36%, respectively. By comparison, two-way focusing in the presence of the obstacle yields relative intensity values of 37%, 21%, 12%, and 9%. Thus, for this scenario the BUS method yields approximately a 4-fold improvement compared to two-way focusing.

## Experimental Results

**Figure 6:**
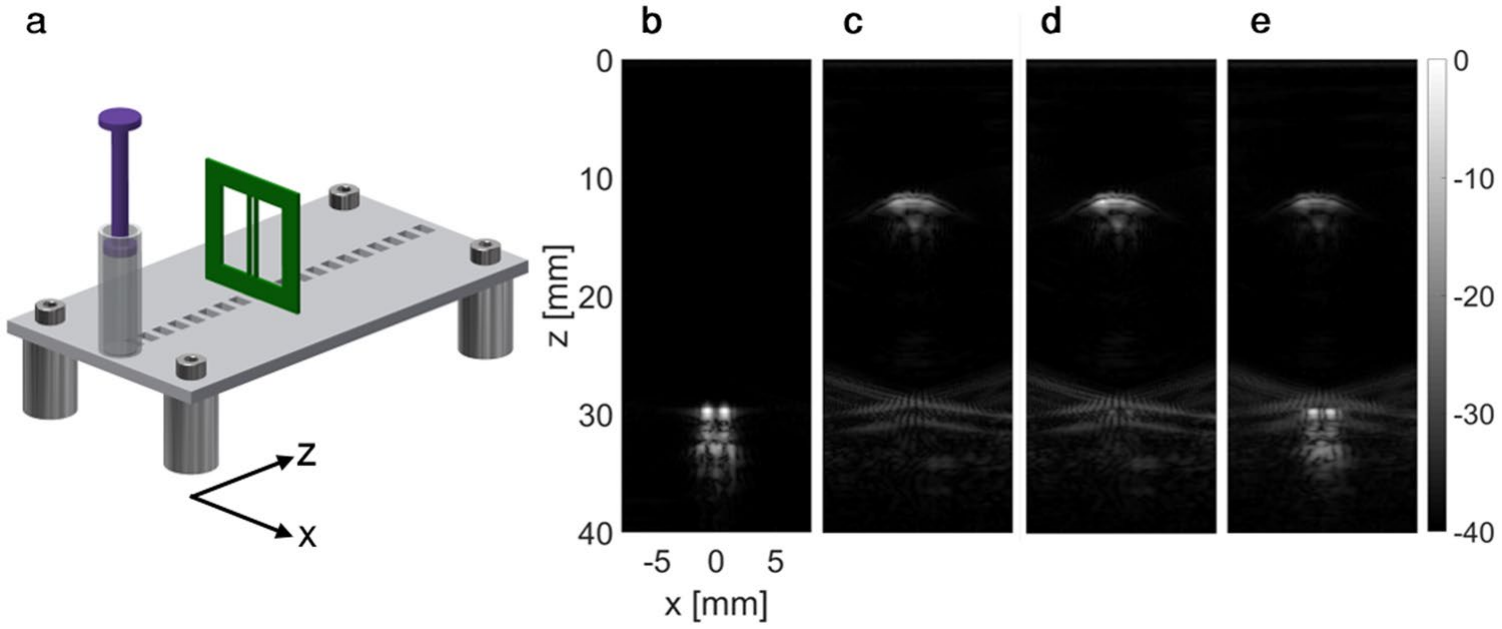
Wire target experimental results. Subfigures are presented with a 40 dB dynamic range. Axes are common to subfigures (**b**–**e**). (**a**) Illustration of the setup used in the experiment. The setup includes two wires and an obstacle, an air-filled syringe, that were placed on a base plate positioned in a water tank and imaged with a transducer. (**b**) A two-way focusing image of the two wire targets (with no obstacle along the beam path). (**c**–**e**) Images of the target in the presence of the obstacle using (**c**) two-way focusing, (**d**) the optimized PI method and (**e**) the BUS method.

To validate the method, we used a target consisting of two acrylic wires with a thickness of 0.7 mm, spaced 1.5 mm apart. The obstacle was a syringe filled with air, positioned between the transducer and the target. The target and the obstacle were placed on an acrylic base plate, positioned in a water tank and imaged with the transducer (Fig. 6a). Initially, a reference two-way focusing image of the target, positioned at a depth of 30 mm, was acquired without the obstacle (Fig. 6b). Next, a 10-mm diameter air-filled syringe was positioned at a depth of 10 mm and a two-way focused image (with full aperture on transmit and receive) was captured, focused at the wire target position. Due to the presence of the air obstacle, the wire target was not detected, and the image is dominated by reflections from the air obstacle (Fig. 6c). Using the optimized PI method, a weak signal from the wires is visible (Fig. 6d). For the BUS method, the patterns of transmission were calculated using the modified Gerchberg–Saxton algorithm to bypass the syringe and generate a focus at the position of the wire target. Fifty shifts of the focal point across the wire target were generated with 0.1 mm steps. The fifty returning echoes were captured and multiplied with the corresponding simulated emitted field. The results were summed and averaged to yield the final reconstructed image of the wire target (Fig. 6e). The similarity between the reference image (Fig. 6b) and the BUS result (Fig. 6e) is notable, providing an initial validation of the method.

**Figure 7:**
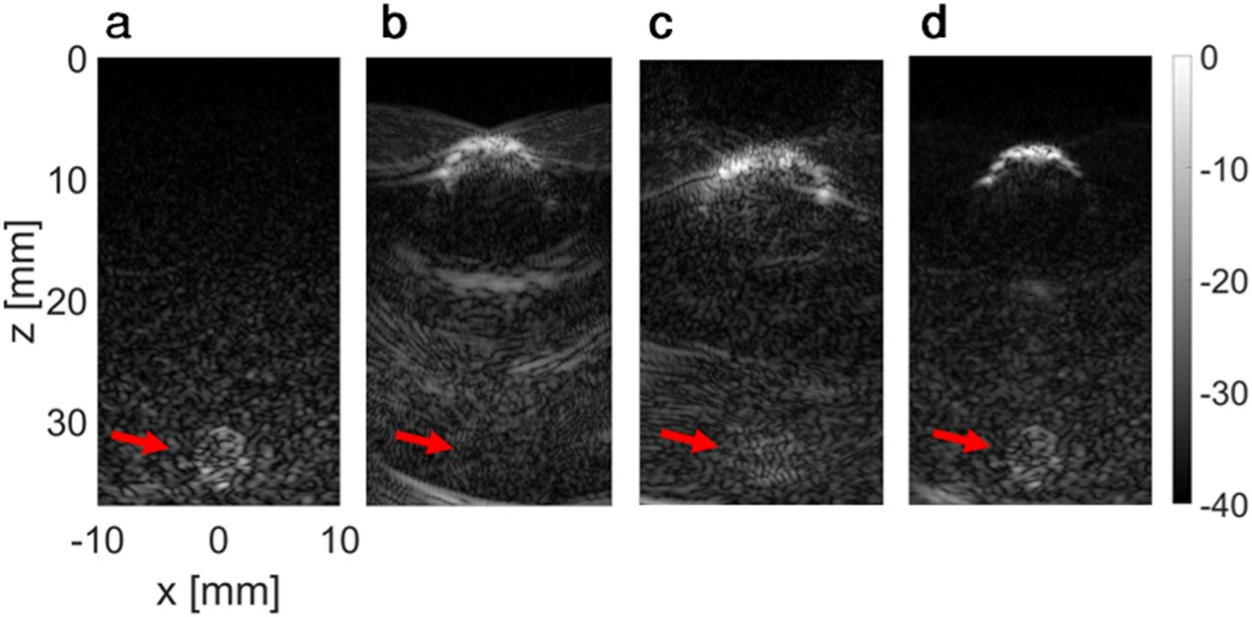
Application of the BUS technique for lesion detection in a tissue mimicking phantom. Images presented in 40 dB dynamic range, and the colorbar is common to all subfigures. Axes are common to subfigures (**a**–**d**). (**a**) Reference two-way focused ultrasound image of the 3-mm lesion positioned at ~32 mm (indicated by the red arrow). (**b**) Two-way focused ultrasound image of the same phantom with a 10-mm air bubble located at a depth of 10 mm. The lesion cannot be seen due the presence of the obstacle. (**c**) Using the optimized PI method, the lesion is dimly visible. (**d**) Using the BUS method, the lesion is fully visible.

Next, an agarose-based tissue mimicking phantom with specular reflectors was used to mimic the detection of small lesions behind an obstacle. Initially, a 3-mm lesion was inserted in the tissue mimicking phantom at a depth of 30 mm, and a two-way focusing image (focused at a depth of 30 mm), was acquired as a reference (Fig. 7a). Next, a gaseous obstacle was formed by drilling a 10-mm hole at 10 mm depth in the tissue mimicking phantom. With two-way focusing (focused at a depth of 30 mm), the lesion is obscured (Fig. 7b). Based on the structural geometry, beam-shaped patterns were generated and used for imaging, similar to the wire experiment. With the optimized PI method, the lesion is detectable (Fig. 7c), however its contrast is only 10% as compared to the reference image (Fig. 7a). Using the BUS method (Fig. 7d), the lesion is detected, with a contrast of 45% as compared to the reference image (Fig. 7a).

**Figure 8:**
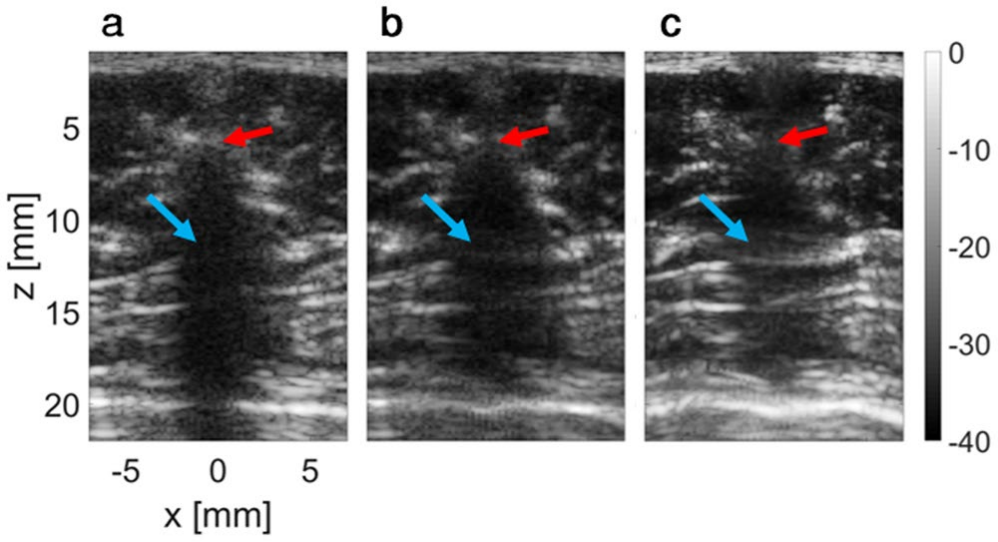
Application of the BUS method in a rat thorax. Images are presented with a 40 dB dynamic range, and the colorbar is common to all subfigures. Axes are common to subfigures (**a**–**c**). (**a**) Standard two-way focused ultrasound image of the area behind the rat spine. The red arrow indicates the position of a vertebra that obscures the tissue behind it (blue arrow). (**b**) Results of the optimized PI method. (**c**) Results of the BUS method, showing a notable improvement in the imaging of the tissue behind the vertebra.

In a third experiment, the thorax of a Sprague-Dawley rat was imaged *ex vivo*, immediately distal to the spine (focused at 15 mm). In Fig. 8a, a two-way focused image, the red arrow marks the position of a spine vertebra, which casts a shadow on the tissue distal to the spine (marked by the blue arrow). The location of the vertebra was estimated based on this image to have a depth of 6 mm and a width of 7 mm. Beam-shaped patterns were generated and imaging was performed similar to the previous experiments both for the optimized PI method (Fig. 8b) and for the BUS method (Fig. 8c). The BUS method yields the clearest image of the tissue distal to the vertebra.

## Discussion

This paper describes a method for visualizing tissue behind obstacles that are otherwise impenetrable to ultrasound. The method uses an optically-inspired holographic algorithm to beam shape the ultrasound emitted field in order to bypass the obstacle and image the area behind it. Through beam shaping, energy from elements that in standard two-way focusing were blocked by an obstacle reaches a region distal to the obstacle. Given the estimated geometry of the image area (obstacle depth, obstacle width and focus depth), which can be obtained using traditional two-way focusing, the BUS algorithm iterates and converges to a solution that generates the desired emitted field pattern. This transmitted pattern is swept across the image, in a manner similar to traditional two-way focusing, to yield high resolution and contrast behind the obstacle. The field of view outside the obstacle is then imaged using standard two-way focusing. Unlike other methods that assume that the obstacles are thin features located immediately in front of the transducer, the proposed method is valid for thick, three-dimensional obstacles. Further, since the phase and apodization maps can change dynamically during transmission, the entire process can be performed in real-time, and the imaging duration is similar to that required for two-way focusing.

Another advantage of the BUS method is that it can be implemented in commercial ultrasound systems without the need for additional components. Further improvement in the contrast of the reconstructed features can be achieved using image-based deconvolution, as suggested in^10^, at the expense of computational complexity.

The performance of the method varies as a function of transducer size, obstacle width and position, as well as the position of the target to be imaged. A 90% focal intensity, relative to the reference two-way focusing without the obstacle, can be generated when the obstacle width is small relative to the transducer width (w/D = ~1/12). With a larger transducer or smaller obstacle, a larger fraction of the emitted field can reach the obstacle using standard two-way focusing, hence the improvement of the BUS method is less significant. The improvement is greatest when w/D > 1/4, and can reach a factor of 30 in intensity and a reduction of a factor of ~3 in the sidelobes.

The positions of both the obstacle and the target also affect the potential focal intensity. For an obstacle close to the transducer (e.g. a depth of 5 mm), a focal intensity reaching 90% of the two-way focusing without obstacle can be achieved, whereas with the same obstacle at a 20-mm depth, a 30% focal intensity is achieved with the same target depth. Also, the focal intensity increases as a function of the separation distance between the obstacle and the target.

The BUS method is designed to image targets distal to obstacles. In the examples presented here, the obstacle is positioned along the central line with respect to the array position. This geometry is ideal because the propagating field bypasses the obstacle equally from each side and the resulting focal spot is symmetrical. When the obstacle is offset from the transducer center line, simulations indicate that an offset of up to 4 mm is tolerated, and in this case the focal intensity is reduced by 10%. Larger offsets in the position of the obstacle can be detected by the user, and the transducer can be realigned accordingly.

Among the uncertainties in the obstacle depth and width, the more significant is the underestimation of the obstacle width. An underestimation of 1 mm reduces the focal intensity by 10%. The tolerance is small because the propagating field bypasses the obstacle in close proximity to the obstacle’s boundaries, to maximize the contribution of all of the elements to the creation of a focal point behind an obstacle. Adding an extra 1 mm to the obstacle width estimation could improve the resulting focal intensity.

When imaging *in vivo*, the choice of the position of the focal spot will be determined by the imaging scene and the position of the actual target. Using the BUS method, a focal point will be generated and is expected to achieve a higher intensity than two-way focusing. We have demonstrated using simulations, wire targets, a tissue-mimicking phantom and tissue samples that the BUS method can be successfully used to image behind ultrasound-impenetrable obstacles such as gas and bones. This increases the potential for ultrasound imaging to be used in currently challenging applications such as in the detection of soft tissue lesions in the presence of abdominal gas^21^, which is important since gas increases the false detection rate for gastrointestinal tumors^22^. Furthermore, intestinal gas limits ultrasound diagnosis for choledocholithiasis^23^, abdominal aortic aneurysms^24^, and hypertrophic pyloric stenosis^25^, and limits the utility of ultrasound findings in Crohn’s disease and ulcerative colitis^26^. Other applications include imaging behind the rib cage for 3D echocardiography^27^, evaluation of intracardiac and extracardiac vascular anatomy^28^ and ultrasound lung imaging^29^. Spinal imaging is another important application: ultrasound is currently used only to outline the spinous vertebrae, as bones reflect most of the ultrasound^30,31^. The BUS method could allow imaging of tissues distal to the spine, opening the door to using 3D ultrasound for evaluating spine deformity^32^, or to enhance ultrasound tomography^33^. Ultrasound imaging in the presence of shunts is also desirable, as shunts are created from materials that interfere with ultrasound propagation^34,35^. In conclusion, we have established the feasibility of applying a beam shaping method to image tissue distal to obstacles that are otherwise impenetrable using ultrasound.

## Methods

### Tissue mimicking phantom preparation

2% agarose powder (Alfa Aesar, MA, USA) and 1.5% silicon carbide for acoustic scattering (HSC1200, d50 = 6 μm, Superior Graphite, Chicago, IL) were mixed with deionized water at ambient temperature and heated until all powder was dissolved, followed by degassing. The degassed solution was poured into an 85 × 70 × 50 mm^3^ mold and cooled until congealed. The speed of sound within this sample was ~1500 m/s^36^.

3-mm rod lesions were prepared similar to the bulk agarose, with 2% silicon carbide poured to the solution after the degassing stage, to generate scattering and change in contrast between the lesion and the background. The degassed solution was poured into 1 mm syringes, with a diameter of 3 mm, and cooled until solidified. A 3-mm diameter hole at a depth of 30 mm was drilled out of the agarose cube, and the rod lesion was inserted in an aqueous environment to prevent air being introduced during the insertion.

The air gap obstacle was generated by drilling a 10-mm diameter hole at 10 mm depth in the agarose, yielding a rod-shaped air gap.

### *Ex vivo* sample preparation

All animal-related work performed by our laboratory was in accordance with the Guide for the Care and Use of Laboratory Animals of the National Institutes of Health (NIH) and all animal experiments were performed under a protocol approved by the Institutional Animal Care and Use Committee (IACUC) of the University of California, Davis. All experiments were performed in accordance with relevant guidelines and regulations. The *ex vivo* sample was from a Sprague-Dawley rat. Imaging was performed perpendicular to the spine of the animal. Prior to the experiment, fur around the imaging area was shaved and then further removed using depilatory cream. The animal was placed in the supine position and the array was positioned using a 3D linear stage. Ultrasound gel was used as coupling agent.

### Computation and ultrasound imaging

The design of the patterns and the post processing of the images were implemented in MATLAB (version 2016b, MathWorks, Natick, MA, USA). The acoustic pressure field corresponding to the calculated phase and apodization maps was simulated using a Matlab code based on the angular spectrum approach and verified using Field II software^37^. Both programs run on a Dell OptiPlex 7040 PC with a Windows 10 Enterprise 64-bit operating system, Intel® Core™ i7-6700 processor, 3.40 GHz, 16 GB RAM. Ultrasound imaging was performed using a programmable ultrasound system (Vantage 256, Verasonics Inc., Kirkland, WA, USA).

Phantom experiments were conducted with a phased array sector transducer P6-3 (ATL Ultrasound Inc., Bothell, WA, USA), at a center frequency of 4.46 MHz. The transducer has 128 elements, with an element size of 0.22 mm and therefore a total aperture of D = 28.2 mm. The transducer position was fixed throughout each experiment.

*Ex vivo* rat experiments were conducted with a compact linear array CL15-7 (Phillips ATL Ultrasound Inc., Bothell, WA, USA), at a center frequency of 8.93 MHz. The transducer has 128 elements, with an element size of 0.18 mm and a total aperture of D = 22.8 mm.

For each experiment, two-way focusing images of the fixed field of view with 256 lines, a line width of 0.1 mm and the same imaging parameters as the BUS method were acquired as reference.

In the proposed method, the location and width of the obstacle and the desired imaging depth are determined based on the two-way focusing reference image. Next, the Gerchberg–Saxton algorithm is then used to calculate the transducer phase and apodization maps, and the emitted fields are simulated to be used in the post processing. A value of 0.95 was used as the convergence threshold, which typically required 20 iteration cycles. Simulation of the transmitted patterns was facilitated using the Verasonics built in Matlab “ShowTXPD” function. This function provides a 2D color encoded display of the transmit beam for every pixel in the ultrasound image field of view, and accounts for the transducer geometry, the medium sound speed and attenuation, and all transmit apodization and waveform parameters. The focal point was shifted between successive transmissions to sweep across the image as in two-way focusing by designing a shifted focal point using the Gerchberg–Saxton algorithm. Next, the transducer was excited with the apodization and phase maps created by the algorithm and received data were recorded for processing. Post processing included the multiplication of each of the transmitted field’s images by the corresponding simulated field, then summing and averaging to achieve the final reconstructed image. A single run of the algorithm with an average of 20 iterations requires ~1 ms on our computer. A full scan of the target using fifty shifts of the focal point therefore introduces a time penalty of 50 ms to the imaging process.

### Data availability

The datasets generated during and/or analyzed during the current study are available from the corresponding author on reasonable request.
